# Proposed Women's Voluntary Hospital Brigade

**Published:** 1914-12-19

**Authors:** 


### MISCONCEPTIONS.

(Correspondence is Invited.)

### PROPOSED WOMEN'S VOLUNTARY HOSPITAL BRIGADE.

The misunderstanding of the proposal put forward by The Hospital to enable women to render the voluntary hospitals practical service at a time of financial stress very aptly illustrates a common disposition of people to overlook duties they are qualified to perform, or to put them aside, in favour of others for whose performance they are unfitted. It is a failing by no means limited to women. Most of us know how often a man capable, and maybe successful, in his own sphere, contrives to persuade himself and attempts to convince his friends of his aptitude for some undertaking quite different and deplores his loss of opportunity to proceed with it.

Possibly the suggested title of the new organisation lent itself to less prosaic conceptions of aim than mere usefulness. At this time of war obsession the notion of a Women's Voluntary Hospital Brigade may have conjured up visions of sick and wounded soldiers whom to tend would be eminently satisfying and interesting, and awakened aspirations only to be very imperfectly fulfilled by helping to pay the institutional bills. The incident encourages the general reflection upon which we have entered, and some good may be effected if it serves to bring home the fact that organised undertakings are rarely, if ever, ready to welcome merely amateur assistance in technical labour. The informed mind finds difficulty in understanding how any intelligent woman wholly untrained supposes she can be a competent nurse in serious cases, or fails to realise that no responsible authority would dream of placing her in charge of sick people, whether soldiers or civilians.

Quite recently Sir Frederick Treves, doubtless with poignant memories of the South African military hospitals at the time of the Boer War, wrote to protest against any repetition of the laxity with which women ignorant of the art of nursing had been allowed to take duty in the wards. There was no sentimental reasoning about the protest; it was concerned entirely with what is due to the patients. Much might be written concerning other aspects of the matter, but that one is chief and all-sufficient. Every doctor, and still more every surgeon, would agree that not seldom the nursing is of first importance; and when the country owes so much to its soldiers, to give their wounded any but the best tendance procurable would be criminal.

